# Effect of manure and mineral fertilisers on the content of light and heavy polycyclic aromatic hydrocarbons in soil

**DOI:** 10.1038/s41598-020-61574-2

**Published:** 2020-03-12

**Authors:** Sławomir Krzebietke, Ewa Mackiewicz-Walec, Stanisław Sienkiewicz, Dariusz Załuski

**Affiliations:** 1 Department of Agricultural Chemistry and Environmental Protection, Faculty of Environmental Management and Agriculture, University of Warmia and Mazury in Olsztyn, Olsztyn, Poland; 2 Department of Plant Breeding and Seed Production, Faculty of Environmental Management and Agriculture, University of Warmia and Mazury in Olsztyn, Olsztyn, Poland

**Keywords:** Environmental monitoring, Geochemistry

## Abstract

A study was conducted to explore the effects of fertilisation with farmyard manure (FYM) and mineral fertilisers on the content of PAHs in soil. The analyses were made on soil samples (collected in 1998–2009) from a long-term field experiment set up in 1986 in Bałcyny near Ostróda. The content of light and heavy polycyclic aromatic hydrocarbons was determined on a gas chromatograph coupled with an FID detector. The analytical data were processed statistically according to an analysis of variance with repeated measurements. The content of light and heavy polycyclic aromatic hydrocarbons was significantly higher in soil fertilised with FYM than in soil nourished only with mineral fertilisers. The effect of increasing doses of potassium on total light PAHs in soil depended on a fertilisation system – there was either a distinct decrease in soil fertilised with mineral substances alone or a slight increase in soil fertilised with manure. Regular soil liming significantly raised the ∑ of heavy PAHs in soil treated with manure but significantly decreased it in soil supplied only mineral fertilisers.

## Introduction

Polycyclic aromatic hydrocarbons (PAHs) are included into the most important environmental pollutants of natural and antropogenic origin including three types of transformation: biological, pyrogenic and petrogenic^1^. It is well documented that PAHs have toxic, carcinogenic and even mutagenic effects on life organisms^1–4^. Although the human activities contributes to the PAH presence in the elevated concentrations in soils, the microorganisms such as bacteria, fungi and algae can successfully participate in the PAH biodegradation^1,5^. The effective way to decontaminate the PAHs including some physical, chemical and biological (bioremediation, phytoremediation) processes can lead to complete mineralization into CO_2_, H_2_O and biomass^6^. Bioavailability of contaminants in soil depends on a number of factors, including the soil properties (content of organic carbon, pH, porosity, composition of the soil solution), characteristics of compounds (molecular structure, solubility in water, volatility, value of the KOW and KOC coefficients) and the biology of organisms (habitat, morphology, age, development stage, viability, etc.) and climate (temperature, moisture) as well as the presence of other compounds^7^. PAHs have a tendency to persist in the environment. PAHs with a low molecular weight are more easily biodegradable than the ones with a higher molecular weight^8,9^. The half-life of low-molecular-weight (LMW) PAHs, e.g. phenanthrene, is from 16 to 126 days, while high-molecular-weight (HMW) PAHs have a longer half-life period, e.g. benzo(a)pyrene can last for up to 1400 days^10^. According to Włóka *et al*.^11^, polycyclic aromatic hydrocarbons with a lower molecular weight are more resistant to biodegradation processes, and their presence is more stable than that of high-molecular-weight PAHs.

PAHs are very mobile, ubiquitous, and a possible grave threat to the environment^12,13^. They are generated as by-products of many chemical processes. The source of these contaminants could be any process involving incomplete incineration of organic compounds. Polycyclic aromatic hydrocarbons present in man’s environment are mostly derived from anthropogenic sources^14^, while the contamination with PAHs from natural sources corresponds to the so-called ‘natural background’. PAHs produced by natural biosynthesis have a less developed structure than the ones from anthropogenic sources^15^.

Several factors may reduce the rate and scope of biodegradation of PAHs in soil^16–21^, e.g. content of organic matter in soil, content of nutrients, fertilisation, the C:N:P ratio, presence and abundance of microorganisms, acceptors of electrons, temperature, moisture, pH and content of oxygen.

The aim of the study was to evaluate the effect of long-term fertilisation with manure and mineral fertilisers on the content of light and heavy PAHs in soil (0–30 cm) during three crop rotation cycles.

## Results and discussion

Organic fertilisers increase the content of organic matter, whereas mineral fertilisation raises the load of biogenic compounds and improves the cation exchange capacity, and liming stabilises the reaction of the soil environment^22^. On the other hand, fertilisers are a potential source of pollutants, including PAHs^11^. The statistical analyses carried out during the study demonstrated a highly significant influence of manure (O) and differentiated mineral fertilisation (M) as well as the interaction of these factors (O x M) on the content of light PAHs (naphthalene, acenaphthene, acenaphthylene, fluorene, anthracene, fluoranthene, pyrene, chrysene) and heavy PAHs (benzo(a)anthracene, benzo(a)pyrene, benzo(b)fluoranthene, benzo(k)fluoranthene, benzo(g,h,i)perylene, indeno(1,2,3-cd)pyrene, dibenzo(a,h)anthracene) in soil (Table 1). Variation in the content of light and heavy PAHs in soil proved to be highly variable in the research years.

**Table 1:** Tow-factorial analysis of variance of the PAHs content in a system with repeated measurements.

Source of variation	df	9 light PAHs	7 heavy PAHs
Manure (O)	1	**	**
Block (B)	2	ns	ns
Mineral fertilisation (M)	7	**	**
O x M	7	**	**
Error 1	30	—	—
Research years (Y)	11	**	**
Y x O	11	**	**
Y x B	22	ns	ns
Y x M	77	**	**
Y x O x M	77	**	**
Error 2	330	—	—

**Level of significance p < 0.01; ns – non-significant; df – degrees of freedom.

### Sum of light PAHs

**Figure 1:**
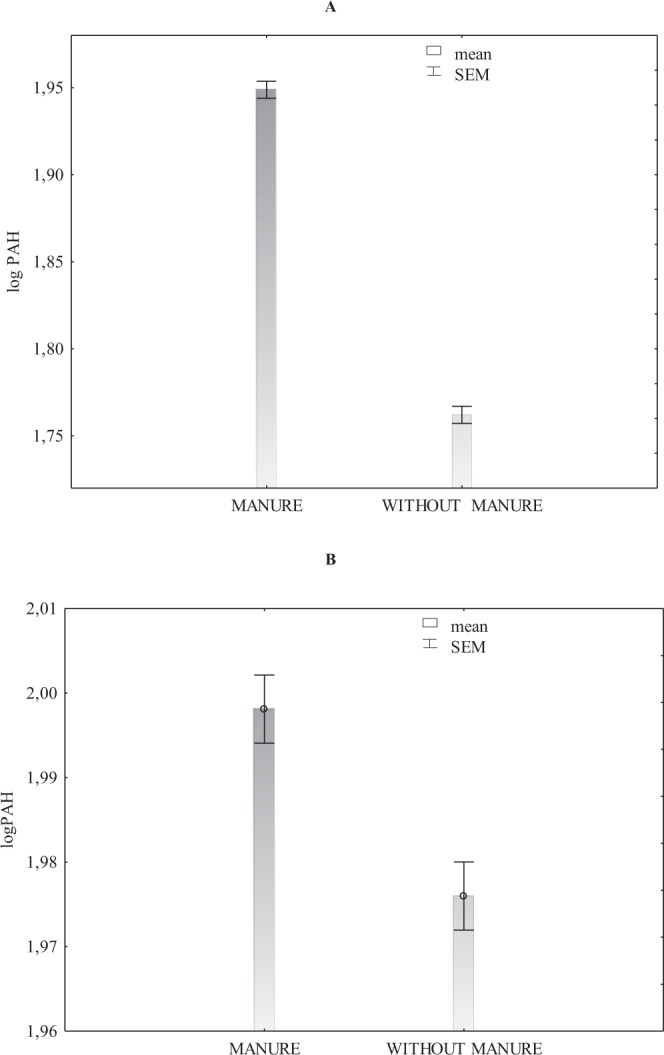
Sum of light PAHs (**A**) and sum of heavy PAHs (**B**) in soil fertilised and not fertilised with FYM (transformed data from the years 1998–2009).

The content of light polycyclic aromatic hydrocarbons (naphthalene, acenaphthylene, fluorene, anthracene, phenathrene, fluoranthene, pyrene, chrysene) was distinctly greater in soil regularly fertilised with FYM (114.2 μg∙kg^−1^ on average) than in soil fertilised only with mineral fertilisers (76.3 μg∙kg^−1^ on average) (Fig. 1A, Table 2).

**Table 2:** Content of the sum of light PAHs in soil in the years 1998–2009 depending on the application of farmyard manure (O), mineral fertilisers (M) and interaction of O x M.

Variants	N_0_P_0_K_0_	N_1_P_1_K_1_	N_2_P_1_K_1_	N_3_P_1_K_1_	N_2_P_1_K_2_	N_2_P_1_K_3_	N_2_P_1_K_2_Mg	N_2_P_1_K_2_MgCa	Mean
**Sum of light PAHs in soil [µg∙kg**^**−1**^**]**									
Manure	97.1	99.0	115.8	112.9	119.1	116.1	119.4	133.9	114.2
Without manure	78.6	87.6	81.4	86.6	71.1	86.0	61.9	57.3	76.3
Mean	87.8	93.3	98.6	99.7	95.1	101.0	90.7	95.6	—

**Figure 2:**
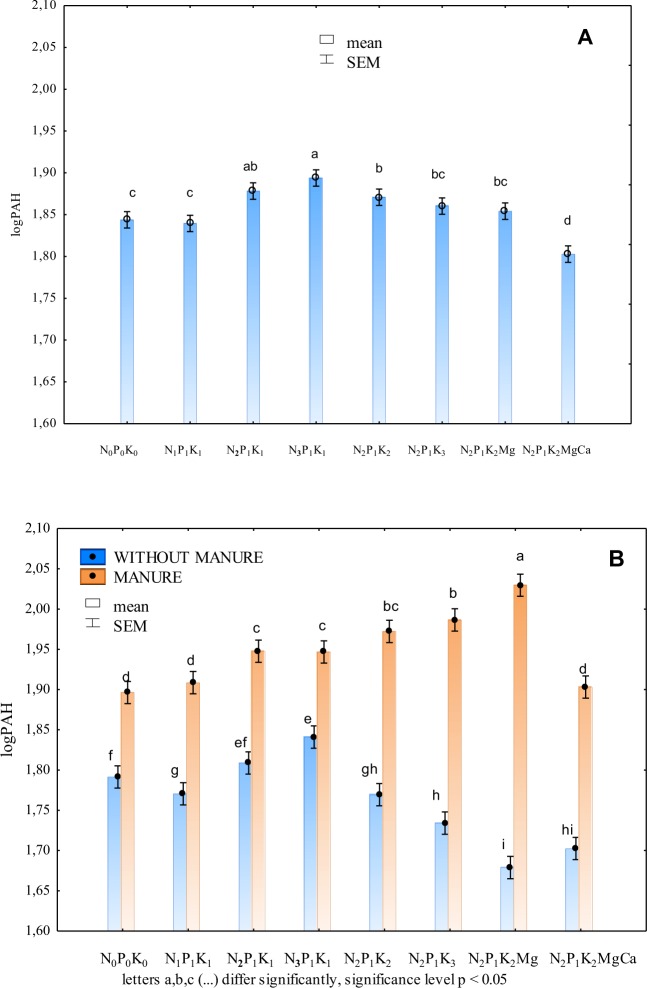
Sum of light PAHs in soil depending on: mineral fertilisation (**A**) and manure-mineral and mineral fertilisation (**B**) (transformed data from the years 1998–2009).

Higher doses of nitrogen significantly increased the content of light polycyclic aromatic hydrocarbons in soil (Fig. 2A, Table 2). A completely reverse effect was produced by potassium, as this element decreased the average content of the analysed substances in soil, and magnesium produced a comparably positive impact. Based on the statistical computations (transformation and elimination of extreme data), it can be concluded that liming is a treatment which leads to a decrease in the soil content of PAHs.

Higher doses of nitrogen contributed to an elevated accumulation of light PAHs in soil fertilised with FYM and with mineral fertilisers alone (Fig. 2A, Table 2). Based on the research results, it can be demonstrated that potassium and magnesium under exclusive mineral fertilisation can limit the accumulation of these contaminants in soil. A reverse effect of the above elements was observed in soil fertilised with FYM and mineral fertilisers (Fig. 2B). Higher doses of nitrogen and magnesium in more fertile soil (nourished regularly with manure) do not create the conditions for microorganisms to use PAHs as a source of energy. Furthermore, a higher supply of available forms of potassium and magnesium in soil receiving only mineral fertilisers can contribute to a higher count of microorganisms. As the microorganisms have less organic matter, they are forced to take advantage of PAHs. It turned out that the calculated average amounts of light PAHs in soil over the period of 12 years are not the best measure to show the variability of this characteristic. It was not until the applied statistical model was employed that a more precise assessment of the soil content of PAHs depending on mineral fertilisation was achievable. On the basis of transformed data, and having eliminated the extreme values, it can be concluded that soil liming under the intensive manure fertilisation conditions can reduce considerably the content of PAHs in soil. No such beneficial effect of liming was observed in soil which received only mineral fertilisation.

### Sum of heavy PAHs

Maliszewska-Kordybach *et al*.^23,24^; Yang *et al*.^25^ and Klimkowicz-Pawlas *et al*.^26^ report on higher concentrations of heavy PAHs than light PAHs in soil. Based on our trials, it is difficult to confirm this dependence, although the mean concentrations calculated for the 12-year-long experimental period showed that the content of heavy PAHs was only slightly higher than that of light ones (Fig. 1A,B, Tables 2 and 3). It was also possible to notice that the content of heavy PAHs in soil fertilised with manure was slightly higher than in soil nourished only with mineral fertilisers (Fig. 1B, Table 3). According to Marquès *et al*.^27^, PAHs with low molecular mass are more rapidly biodegraded when the temperature and light intensity increase. Similar results were reported by Park *et al*.^8^. In a study carried out by Mazur *et al*.^28^, the most abundant PAHs in soil were the three-ringed ones. Also, Włóka *et al*.^11^ as well as Zhao *et al*.^29^ demonstrated the dominance of 2- and 3-ringed PAHs.

**Table 3:** Content of the sum of heavy PAHs in soil in the years 1998–2009 depending on the application of farmyard manure (O), mineral fertilisers (M) and interaction of O x M.

Variants	N_0_P_0_K_0_	N_1_P_1_K_1_	N_2_P_1_K_1_	N_3_P_1_K_1_	N_2_P_1_K_2_	N_2_P_1_K_3_	N_2_P_1_K_2_Mg	N_2_P_1_K_2_MgCa	Mean
**Sum of heavy PAHs in soil [µg∙kg**^**−1**^**]**									
Manure	115.1	97.8	127.4	137.0	127.8	109.0	119.4	127.2	120.1
Without manure	93.3	102.9	108.4	128.3	112.2	124.1	129.4	98.6	112.2
Mean	104.2	100.4	118.0	132.7	120.1	116.5	124.4	112.9	—

**Figure 3:**
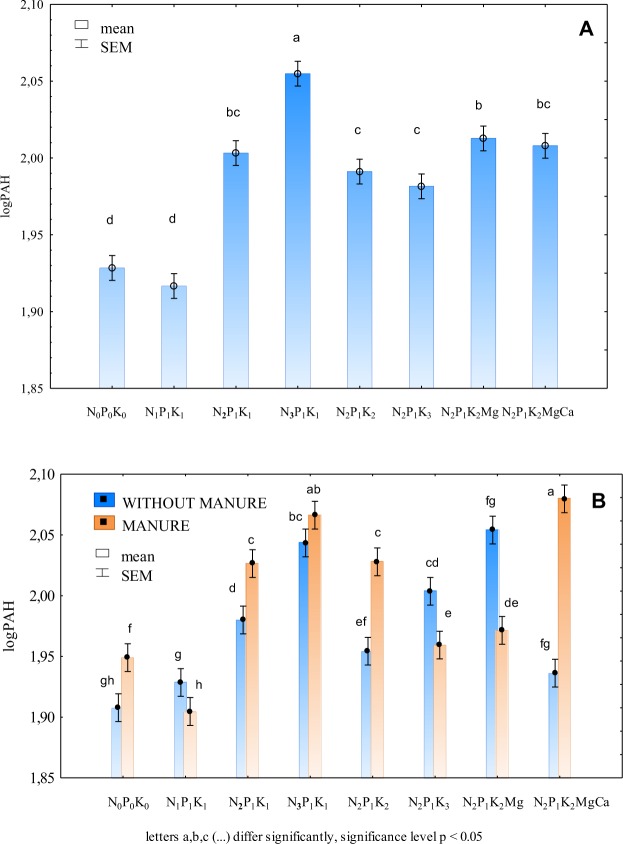
Sum of heavy PAHs in soil depending: on mineral fertilisation (**A**) and manure-mineral and mineral fertilisation (**B**) (transformed data from the years 1998–2009).

The content of total heavy PAHs (benzo(a)anthracene, benzo(a)pyrene, benzo(b)fluoranthene, benzo(k)fluoranthene, benzo(g,h,i)perylene, indeno(1,2,3-cd)pyrene, dibenzo(a,h)anthracene), similarly to the sum of light PAHs, increased under the influence of larger doses of nitrogen (Fig. 3A, Table 3). The highest soil content of heavy PAHs (132.7 µg∙kg^−1^) was determined after the application of the highest nitrogen dose. Potassium did not have any significant effect on the soil content of PAHs. Regular soil liming had a positive effect, diminishing the content of the above pollutants in soil.

Changes in the content of heavy PAHs in soil depending on mineral fertilisation carried out with or without manure ran a completely different course than those concerning light PAHs (Fig. 3B, Table 3). The difference was the greatest in limed soil. Soil liming carried out on soil fertilised exclusively with mineral fertilisers very strongly reduced the content of heavy PAHs in soil, while having an opposite effect in soil fertilised also with farmyard manure. In addition, lower amounts of PAHs were determined in soil regularly fertilised with manure and the highest dose of potassium or N_2_P_1_K_2_Mg than in soil fertilised only with mineral fertilisers. Same as for light PAHs, the content of heavy PAHs in soil was strongly affected by nitrogen. In the soil fertilised only with mineral fertilisers with a dose of nitrogen, the content of heavy PAHs increased proportionally. It is possible to conclude that manure had a weaker effect on the content of heavy PAHs than on the content of light PAHs in soil.

According to Regulation of the Minister of the Environment of September 1, 2016 on the method of conducting soil pollution assessment (Journal of Laws of 2016, item 1395)^30^, in Poland is an obligation to identify 10 compounds from the PAH group: naphthalene, anthracene, chrysene, benzo(a)anthracene, benzo(a)pyrene, benzo(b)fluoranthene, benzo(k)fluoranthene, benzo(g,h,i)perylene, indeno(1,2,3-cd)pyrene, dibenzo(a,h)anthracene on an arable land in the 0–30 cm layer. The content of the sum of the above mentioned compounds should not exceeded 1400 µg∙kg^−1^ of soil. According to the IUNG (Institute of Soil Science and Plant Cultivation) classification Kabata–Pendias *et. al*.^31^ and Siebielec *et. al*.^32^ proposed the division of pollutant content of the ∑16 PAH as follows: below 200 µg∙kg^−1^ of soil as natural content, 200–600 µg∙kg^−1^ of soil as elevated but not contaminated soil, whereas the range of 600–1000 µg∙kg^−1^ of soil as low-contaminated at which content the restrictions on plant cultivation should be considered, especially for children and infants. An assessment of long-term use of natural fertilisers (manure) at a dose of 40 t∙ha^−1^ every two years as a source of PAH in soil, despite a slight increase in the ∑16 PAH compared to mineral fertilisation, is safe for the soil environment if it is the only source of these pollutants. When assessing the average level of PAH contaminations in soils fertilised with both natural and mineral fertilisers, there were no exceedances of the assessed compounds that could have a negative impact on increasing their share in soil above the applicable standard content.

## Conclusions

Regular application of large doses of manure (40 t ha^−1^ every two years) can raise the load of PAHs in soil. In our study, the content of light and heavy PAHs was higher in soil fertilised with manure than in soil fertilised only with mineral fertilisers. The impact of increasing doses of potassium on the sum of light PAHs in soil depended on the fertilisation regime – there was a distinct decrease in soil fertilised only with mineral fertilisers and a slight increase in soil fertilised with manure. Regular soil liming significantly increase the content of the ∑ of heavy PAHs in soil fertilised with manure while decreasing it significantly in soil fertilised only with mineral fertilisers. However, lime is not a PAH carrier, it was used only to maintain the proper soil pH, i.e. slightly acidic conditions (pH 5.5–6.6) which, in turn, were more favorable for e.g. faster mineralization of the organic matter. Probably, bacteria could then use more easily available food compounds from the manure used, instead of use hardly available carbon from PAHs.

## Material and methods

### Description of the field experiment

The experiment was set up in 1986, in the village Bałcyny near Ostróda, the Province of Warmia and Mazury in Poland. The experiment was established according to the design described in Table 4, with three replications (blocks), on grey-brown podzolic soil developed over light loam and classified in the Polish taxonomy as class IIIa, very good rye complex (Haplic Luvisols, IUSS Working Group WRB^33^). Based on the particle size distribution, the soil was classified as sandy loam according to United States Department of Agriculture (USDA).

**Table 4:** Design of the field trial.

Series with FYM			Series without FYM		
Block (repeat)	Plot number	Mineral fertilisation (Table 5)	Block (repeat)	Plot number	Mineral fertilisation (Table 5)
I	1	3	I	48	4
	2	7		47	6
	3	4		46	3
	4	1		45	5
	5	8		44	1
	6	2		43	2
	7	6		42	8
	8	5		41	7
II	9	4	II	40	5
	10	6		39	8
	11	7		38	6
	12	1		37	3
	13	8		36	7
	14	2		35	2
	15	3		34	4
	16	5		33	1
III	17	1	III	32	8
	18	7		31	5
	19	8		30	7
	20	2		29	4
	21	4		28	1
	22	6		27	5
	23	3		26	2
	24	5		25	6

The experiment comprised fertilisation with farmyard manure (from cows) and mineral fertilisers or with mineral fertilisers alone. Doses of nutrients in mineral fertilisers (ammonium nitrate (N), triple granuled superphosphate 46% (P_2_O_5_), potassium salt 60% (K_2_O) lime (CaO), kizerite 27% MgO) were on the same levels in both fertilisation regimes. Crops were cultivated in the following rotation sequence: sugar beet, spring barley, maize, and spring wheat. The mineral fertilisation regime is presented in Table 5. Prior to the experiment, 1 kg of soil contained available nutrients: 100.0 mg K, 53.2 mg Mg, 41.3 mg P, 7.9 g organic carbon, and 0.79 g total nitrogen, while the soil reaction was slightly acid at pH KCl (1 mol·dm^−3^) = 6.2. Lime in the form of CaO was applied in an amount of 2.5 t∙ha^−1^ every four years, after harvesting spring wheat (variant 8). Farmyard manure was applied in a dose of 40 t∙ha^−1^ every other year, under sugar beet and maize. Spring barley and spring wheat were grown a year after the application of FYM. The content of mineral components and PAHs in FYM were presented in Table 6.

**Table 5:** Mineral fertilisation regime.

No	Variant	Sugar beet *Spring barley* Maize Spring wheat*			
		N	P	K	Mg
		Dose [kg∙ha^−1^]			
1	N_0_P_0_K_0_	0 0 **0** 0*	0* 0	0 0 **0** 0*	0 0* **0**
2	N_1_P_1_K_1_	60 30 **60** 40*	34.9* 26.2	66.4 *33.2* **49.8** 24.9*	0 0* **0**
3	N_2_P_1_K_1_	120 *60* **120** 80*	34.9* 26.2	66.4 *33.2* **49.8** 24.9*	0 0* **0**
4	N_3_P_1_K_1_	180 90 **180** 120*	34.9* 26.2	66.4 *33.2* **49.8** 24.9*	0 0* **0**
5	N_2_P_1_K_2_	120 60 **120** 80*	34.9* 26.2	132.8 *66.4* **99.7** 49.8*	0 0* **0**
6	N_2_P_1_K_3_	120 60 **120** 80*	34.9* 26.2	199.3 *99.7* **149.7** 74.7*	0 0* **0**
7	N_2_P_1_K_2_Mg	120 60 **120** 80*	34.9* 26.2	132.8 *66.4* **99.7** 49.8*	48.2 18.1* 24,1
8	N_2_P_1_K_2_MgCa	120 60 **120** 80*	34.9* 26.2	132.8 *66.4* **99.7** 49.8*	48.2 18.1* 24,1

**Table 6:** The mean mineral composition and content of PAHs in farmyard manure.

Mineral composition in FYM					
macroelements		microelements		metals	
g∙kg^−1^ DM (dry matter)		mg∙kg^−1^ DM			
N (nitrogen)	17.5	Cu (copper)	36.8	Cd (cadmium)	0.26
P (phosphorous)	6.7	Zn (zinc)	223	Hg (mercury)	0.06
K (potassium)	11.4	Co (cobalt)	5	Pb (lead)	<2.50
Mg (magnesium)	6.1	Mn (manganese)	334	Cr (chromium)	3.51
Ca (calcium)	18.2	Mo (molibdenium)	<5.00	Ni (nickiel)	6.64
S (sulphur)	4.9				
**Content of PAHs in FYM**					
**light**		**heavy**		**sum**	
**µg∙kg**^**−1**^ **DM**					
NAP (naphthalene)	<10.0	(BaA) benzo(a)anthracene	25	∑16 PAHs − 307.0	
ACE (acenaphthene)	<10.0	(BaP) benzo(a)pyrene	21		
ACY (acenaphthylene)	<10.0	(BbF) benzo(b)fluoranthene	45		
FLU (fluorene)	<10.0	(BkF) benzo(k)fluoranthene	22		
ANT (anthracene)	<10.0	(BghiP) benzo(g,h,i)peryleme	16		
PHN (phenanthrene)	40	(InP) indeno(1,2,3,-cd)pyrene	18		
FTH (fluoranthene)	59	(DahA) dibenzo(a,h)anthracene	10		
PYR (pyrene)	37				
CHR (chrysene)	24				

### Analytical methods

The research material consisted of soil samples collected in 1998–2009, from a long-term, controlled field experiment, carried out in Bałcyny since 1986. The soil was sampled with a soil sampler, each time obtaining around 1 kg of soil. Having been dried to the air-dry state, i.e. subjected to dry-air drying at room temperature, each soil sample was sifted through a 2 mm mesh sieve.

The content of heavy and light molecular weight polycyclic aromatic hydrocarbons was determined on a gas chromatograph – mass spectrometer Trace GC Ultra ITQ900 coupled with an autosampler TRIPlus (Fisher Scientific) manufactured by THERMO, and equipped with an FID detector. An analysis of the content of polycyclic aromatic hydrocarbons (PAHs) was accomplished after one-hour extraction of 20 g of soil with 20 cm^3^ of acetonitrile, using an ultrasound washer and horizontal shaker. The extract thus obtained (10 cm^3^) was decanted and preliminarily purified on an MPW-350R centrifuge and a solid phase extract SPE station. SPE-NH_2_/C18 cartridges with the adsorbent weight of 1500 mg and the capacity of 6 cm^3^ were used. 10 cm^3^ of methanol was applied to flush the PAHs from the adsorbent, after which the extract was concentrated in a neutral gas (nitrogen) atmosphere up to the volume of 0.2 cm^3^. The samples prepared as described above were subjected to determinations of PAHs with the GC technique, using an FID detector mounted on an Rxi-5ms column 30 m in length, and the inner diameter of 0.25 mm, where the walls were coated with a 0.25-µm thick layer of liquid stationary phase (SCOT column technology). He at a constant flow rate (3 cm^3^∙min^−1^), and H_2_, air and N_2_ at the respective flow rates of 35, 350 and 30 cm^3^∙min^−1^) served as carrier gases. The temperature regime was as follows: 0–100 °C – 0.2 min; 50 °C∙min^−1^ – 143 °C – 1.5 min; 8 °C∙min^−1^ – 180 °C – 0.4 min; 100 °C∙min^−1^ – 210 °C – 1.5 min; 10 °C∙min^−1^ – 300 °C – 5 min = 23.39 min. The temperature of detectors was set at 340 °C, while the temperature of the splitless injector was set at 250 °C. Determinations were made based on the reference solution by Restek Corporation, containing a mix of 16 PAHs (naphthalene, acenaphtylene, fluorene, anthracene, phenathrene, fluoranthene, pyrene, chrysene, and a sum of heavy PAHs: benzo(a)anthracene, benzo(a)pyrene, benzo(b)fluoranthene, benzo(k)fluoranthene, benzo(g,h,i)perylene, indeno(1,2,3-cd)pyrene, dibenzo(a,h)anthracene in a concentration of 2000 µg∙cm^−3^ of each component compound. Working solutions equalled 5, 10, 20, 50, 80, 120 µg∙cm^−3^ of each of the components. The recovery of PAHs from soil ranged from 84% to 93%, and was considered separately for each of the compounds analysed.

The sum of light PAHs was composed of naphthalene, acenaphthene, acenaphthylene, fluorene, anthracene, phenathrene, fluoranthene, pyrene and chrysene. The sum of heavy PAHs was made up of benzo(a)anthracene, benzo(a)pyrene, benzo(b)fluoranthene, benzo(k)fluoranthene, benzo(g,h,i)perylene, indeno(1,2,3-cd)pyrene, dibenzo(a,h)anthracene.

### Statistical analysis methods

Statistical processing of the data^34^ was based on an analysis of variance with replicated measurements–3 replications (blocks–B), where two factors: ‘FYM fertilisation’ (O) and ‘differentiated mineral fertilisation’ (M) were treated as constants and aggregating, while ‘years’ (Y; *Years*) was seen as a factor of the repeated measurements which was identified (involved in) the effect ‘crop species’.

$${y}_{ijkl}=\mu +{\tau }_{i}+{f}_{k}+{(\tau f)}_{ik}+Year{s}_{l}+{(\tau Years)}_{il}+{(fYears)}_{kl}+{(\tau fYears)}_{ikl}+{\beta }_{j}+{(\beta Years)}_{jl}+{\varepsilon }_{ijkl}$$

where: *μ* is general mean, *τ*_*i*_– effect of applying NPK fertilisation and, *f*_*k*_ – FYM fertilisation k, *β*_*j*_ – effect of a block j, Years_l_ – effect of years of the experiment as a factor of repeated measurements, ${(\tau f)}_{ik}$– effect of interaction of the i^th^ level of NPK fertilisation with k – FYM fertilisation, ${(\tau Years)}_{il}$ – effect of interaction of the i^th^ level of NPK fertilisation with l^st^ year of measurements, ${(fYears)}_{kl}$ – interaction of k – FYM fertilisation with 1st year of measurements, ${(\beta Years)}_{jl}$– effect of interaction of ith level of NPK fertilisation with k FYM fertilisation against the background of 1st year of measurements, ${\varepsilon }_{ijkl}$ – random experimental error with normal distribution and with the expected value equal zero and variance σ^2^.

Prior to making statistical analyses according to the developed model, the assumption of normal distribution of variables within each group was tested. Next, homogeneity of variance in groups was analysed, and additional requirements were accounted for, such as an assessment of sphericity, i.e. the equality of variances of the differences between the measurements. The hypothesis of sphericity was verified by the Mauchley’s test. When the violation of sphericity was detected, the multidimensional Wilks’ test and Pilai’s test were performed. Having conducted the Shapiro-Wilks’ test, the assumption of normality of the analysed characteristics was discarded, which led to the application of logarithmic transformation. At the subsequent stage of statistical data processing, *post-hoc* comparisons were made using the Tukey’s test (HSD) at p < 0.05. All statistical tests were supported by the software STATISTICA (StatSoft, Inc., 2014).
